# Antihyperglycemic, antihyperlipidemic and hepatoprotective effects of *Ficus ottoniifolia *(Miq.) Miq. supplementation in alloxan-induced diabetic rats 

**DOI:** 10.22038/AJP.2020.16958

**Published:** 2021

**Authors:** Chinyere Aloke, Ngwu Nwachukwu, Nwogo Ajuka Obasi, Chinedum Uche Emelike, Pascal Anyaegbunam Amu, Patience Nkemjika Ogbu, Onyebuchi Federick Orinya, Egwu Chinedu Ogbonnia

**Affiliations:** 1 *Department of Medical Biochemistry, Faculty of Basic Medical Sciences, Alex Ekwueme Federal University, Ndufu-Alike, Abakaliki, Nigeria*; 2 *Department of Biochemistry, Faculty of Sciences, Federal University of Technology, Owerri, Imo State, Nigeria*; 3 *Department of Physiology, Faculty of Basic Medical Sciences, Alex Ekwueme Federal University, Ndufu-Alike, Abakaliki, Nigeria*; 4 *Department of Science Laboratory Technology, Institute of Management Technology, Enugu, Enugu State, Nigeria*; 5 *Department of Medical Biochemistry, Faculty of Basic Medical Sciences, Ebonyi State University, Abakaliki, Nigeria*

**Keywords:** Alloxan, Diabetes, Ficus ottoniifolia, Hepatoprotective Hyperglycemia, Supplemented diet

## Abstract

**Objective:**

Medicinal plants provide better and cheaper alternative therapy for management of several diseases compared to orthodox medicines. This study evaluated the effects of feed formulated with *Ficus ottoniifolia *(Miq.) Miq. (FFFO) leaves in the management of alloxan-induced diabetes mellitus (DM) in rats.

**Materials and Methods:**

DM was induced in overnight-fasted rats by administration of alloxan monohydrate intraperitoneally. DM rats in Groups 1−3 were fed with graded FFFO while group 4 (diabetic control) and group 5 (normal control) were fed with commercial feeds (Vital-Feeds), daily for 21 days. Changes in body weight and some biochemical parameters were thereafter determined.

**Results:**

Results showed significant decreases (p<0.05) in serum high-density lipoprotein (HDL) but significant increases (p<0.05) in blood glucose, serum low-density lipoprotein (LDL), triglyceride (TG), total cholesterol (TC) and alanine aminotransferase (ALT) activities in DM-induced rats compared to the normal control group. Feeding with FFFO significantly increased (p<0.05) the body weight and HDL, decreased the blood glucose, serum LDL, TG and TC and attenuated ALT activities in FFFO-fed DM rats compared to the diabetic control group.

**Conclusion:**

This study revealed that FFFO-diet may mitigate hyperglycemia, dyslipidemia and liver-damage associated with DM.

## Introduction

Diabetes mellitus (DM) is one of the commonest metabolic ailments connected with micro and macro vascular problems that culminate in profound diseased state and death. It is included among the five prime conditions that result in death in the world (Kumar et al., 2006 ▶). There is no effective therapy available in modern medicine forthe management of DM (Sumana and Suryawashi, 2001 ▶). Considering the negative effects resulting from the usage of insulin and other agents that decrease blood glucose, the demand for use of natural products with anti-diabetic potentials has increased (Rao et al., 2010 ▶). 

The liver performs more functions than any other organ. It is apparent that any disease state or severe pathological conditions that affect the hepatocytes, will result in coordinated and huge metabolic disorders (Aja et al., 2013 ▶). If there is injury or damage to cell membranes in tissues, cell-bound enzymes will be possibly released into the blood and an elevated level of specific enzymes is usually associated with specific tissue or organ damage. Although liver enzymes are present in body tissues, their high levels are most often linked with hepatic injury (Bamidele et al., 2014 ▶). Injection of alloxan to rats and many other kinds of animals brings about damage, particularly to the β cells of the pancreas where insulin is synthesized (Lenzen, 2008 ▶). This damage results in biochemical alterations characteristic of DM with features akin to type1 diabetes seen in humans (Szkudelsk, 2001 ▶). 

Medicinal plants are increasingly emerging as a substitute and/or complimentary therapy in the treatment of DM. The high cost and debilitating side effect of anti-diabetic drugs are clinical challenges that warrant dietary approach in diabetes management (WHO, 2002 ▶). Plant explorations to determine their beneficial biological activities are producing insights into alternative natural products for management of several diseases including diabetes (Jung, 2006 ▶). 

*Ficus ottoniifolia* is a medium-sized plant called “Ogbu” by the Igbo people of South Eastern Nigeria. Its English name is “hedge fig plant”. The leaves of this plant have many medicinal uses. The antioxidative potential of feed formulated with the leaves of *Ficus ottoniifolia*(FFFO) has been reported in the literature (Idenyi et al., 2009 ▶). Literature has revealed that phenolic compounds present in some vegetables and fruits were responsible for their antioxidant activity (Wang et al., 1999 ▶). The leaves of *F. ottoniifolia* are utilized in preparation of soup (Oselebe et al., 2012 ▶). There is paucity of information in literature on the pharmacological effects of this plant, but it has been reported that *F. ottonifolia* is used as laxative and galactagogue (Hamill et al., 2000). To our knowledge, the antihyperglycemic, antihyperlipidemic and hepatoprotective potentials of *F. ottoniifolia* leaves remain to be reported. Thus, the current study investigated the antihyperglycemic, antihyperlipidemic and liver protective activity of FFFO in diabetic rats.

## Materials and Methods

**Chemicals**

Alloxan monohydrate used in this study was sourced from Sigma-AldrichChemical Company of Saint Loius, Missouri, USA*.* RANDOX commercial kits were employed for biochemical analysis. All other chemicals employed were of standard grade. 

**Experimental animals**

Seven-week old Wistar rats that weighed between 105 and 162 g sourced from animal facility of the Faculty of Pharmaceutical Sciences, University of Nigeria, Nsukka, were used. Shelter was provided for the rats in the animal facility of the Department of Biochemistry, Ebonyi State University, Nigeria, under standard conditions (25^o^C and 12 hr light/12 hr dark cycle). The 25 rats were randomly divided into five groups (n=5). They were kept in cages containing wood-chip bedding and allowed free access to standard pellet diet (Vital Feeds Nigeria Ltd, Jos, Nigeria) and clean water, *ad libitum*. Acclimatization for the rats was done for seven days prior to the treatment and the rats were carefully managed in accordance with the approved standard protocol of NIH Guidelines for the Care and Use of Laboratory Animals (NRC, 1985). Ethical approval for this study was granted by the Faculty of Basic Medical Sciences of Ebonyi State University, Abakaliki, Ebonyi State, Nigeria (No: EBSU/FBMS/RE/2016//05/01/001).

**Preparation of experimental feed**

*Ficus ottoniifolia *leaves were sourced from an arable land in a remote village of Ugwulangwu in Ohaozara Local Government Area of Ebonyi State. A plant taxonomist, Mr Alfred Ozioko of the International Centre for Ethnomedicine and Drug Development Nsukka, Enugu State, carried out the identification and authentication of the plant. The voucher specimen (InterCEDD/16288-ficus) has been deposited in the herbarium. The leaves were air-dried to a constant weight and pulverized. Thereafter, the pulverized form was sieved to obtain its powdered form. The powdered form was stored in polyethylene bags maintained at room temperature. Thereafter, the feed was formulated by blending the powdered leaves with the normal rat feed (Vital Feeds Nigeria Ltd, Jos, Nigeria) at 10, 15 and 20% w/w with little addition of water. This was manually made into pellet form followed by drying to a constant weight.

**Experimental design**

The rats were grouped into five, after one week of acclimatization. Diabetes was induced in fasting rats (groups 1 to 4) by intraperitoneal (IP) injection of alloxan (200 mg/kg bw) dissolved in normal saline (Richard et al. 1983 ▶). The fasting blood glucose level was determined using a glucometer (ACCUTREND GC Boehringer, Mannheim) three days after diabetes induction and after treatment. The groups are as follows:

Groups 1-3: diabetic rats + 10% of FFFO, 15% FFFO, or 20% FFFO, respectively.

Group 4 (diabetic control): diabetic rats thatonly received the rat feed (Vital Feeds Nigeria Ltd, Jos, Nigeria), 

Group 5 (normal control): non-diabetic rats that only received the rat feed.

The experiment lasted for 21 days and the rats were humanely anesthetized using diethy ether. Through cardiac puncture, blood samples were collected into sterile bottles. Thereafter, the samples were centrifuged (3000×g for 15 min) and serum was obtained for biochemical investigations.

**Determination of lipid profile parameters and** **liver function assays**

The serum concentrations of total cholesterol (TC), triglyceride (TG), high-density lipoprotein-cholesterol (HDL), low-density lipoprotein (LDL) and very low-density lipoprotein (VLDL), total protein (TP), and albumin (ALB) as well as the activities of alanine aminotransferase (ALT), aspartate aminotransferase (AST) alkaline phosphatase (ALP) and lactate dehydrogenase (LDH) were determined spectrophotometrically in accordance with the manufacturer’s instructions, using commercially available kits procured from Randox. 

**Statistical analysis**

Analysis of data was carried out using the IBS-SPSS (version 20) statistical software (IBM, Corp., Atlanta, GA) and results are presented as mean±standard deviation. Parameters were compared among groups using one-way analysis of variance (ANOVA) and Tukey *post-hoc *test. Statistical differences were established at p<0.05.

## Results

**Effect of FFFO on fasting blood glucose and weight of alloxan-induced diabetic rats**

The effects of FFFO on fasting blood glucose and weight of alloxan-induced diabetic rats, are shown in Table 1(A-B).FFFO prominently decreased (p<0.05) the blood glucose level in comparison to the diabetic control group. However, the lowest level for reduction was observed for15% of FFFO (group 2, Table 1A). FFFO markedly (p<0.05) improved the body weight of treated diabetic rats compared to the diabetic control group. The improvement in body weight by the feed was more pronounced with 15% FFFO (Table 1B).

**Table 1A:** Effect of FFFOleaves on fasting blood glucose of alloxan-induced diabetic rats.

**Blood glucose (mg/dl)**	**DR + 10% FFFO**	**DR + 15 % FFFO**	**DR + 20 % FFFO**	**DC**	**NC**
Initial	173.0±1.3	174.0±0.2	176.0±0.1	151.0±0.3	77.0±1.1
Final	133.0±1.1*	99.0±0.2*	116.0±1.0*	180.0±1.3^#^	85.0±1.2
%change inglucose	-40 (23%)	-75 (43%)	-60 (34%)	+29 (19.2%)	8 (10.4%)

**Table 1B:** Effect of FFFO leaves on body weight of alloxan induced diabetic rats.

**Body weight (g)**	**DR+10% FFFO**	**DR+15% FFFO**	**DR+20% FFFO**	**DC**	**NC**
Initial	130.0±1.5	144.0±1.3	116.0±1.4	160.0±1.2	110.0±0.1
Final	145.0±2.0*	158.0±1.1*	151.0±2.0*	134.0±1.0^#^	144.0±1.3
Change in weight (%)	+15 (10.3%)	+14 (9.7%)	+35 (30.2 %)	-26 (16.3%)	+34 (30.9%)

Values are mean±standard deviation. *p<0.05 shows a significant difference when compared with the diabetic control (DC) #p<0.05 shows a significant difference when compared with the normal control (NC). DR +10% FFFO: diabetic rats that received with 10% feed formulated with *Ficus ottoniifolia *(FFFO) leaves; DR + 15% FFFO: diabetic rats that received 15% FFFO; DR + 20% FFFO: diabetic rats that received 20% FFFO; DC (diabetic control): diabetic rats that received the rat feed (Vital Feeds), NC (normal control): non-diabetic rats that received the rat feed (Vital Feeds). % change glucose = Final minus Initial glucose/initial glucose x 100; %change weight= Final minus Initial weight/initial weight x 100.

**Table 2:** Effect of FFFO on serum liver enzymes (IU/L), ALB (g/L) and TP (g/L) in diabetic rats

**Parameters**	**DR+10% FFFO**	**DR+15% FFFO**	**DR+20% FFFO**	**DC**	**NC**
LDH (IU/L)	189.00±1.00	103.00±2.00*	111.00±1.00*	167.00±45.75	140.00±41.68
ALP (IU/L)	66.00±1.30*#	71.00±1.00*	92.00±1.00	103.75±11.07	101.00±18.77
ALT (IU/L)	7.70±0.10*	7.20±0.10*	6.90±0.10*	9.78±1.03#	7.05±1.00
AST (IU/L)	6.60±0.10*#	7.40±0.20*#	7.00±1.00*#	5.40±0.39	5.38±0.74
TP (g/L)	5.10±0.00	4.80±0.80	5.30±0.20	5.24±0.45	5.68±0.57
ALB (g/L)	3.70±1.30	3.10±1.10	3.10±0.10	3.10±0.73	4.30±0.32

Values are mean±standard deviation (n=5) *p<0.05 shows a significant difference when compared with the diabetic control (DC); #p<0.05 shows a significant difference when compared with the normal control (NC). DR+10% FFFO rats was fed with 10% feed formulated with *Ficus ottoniifolia *(FFFO); DR+15% FFFO was fed with 15% FFFO; DR+20% FFFO was fed with 20% FFFO. DC (diabetic control): diabetic rats that received the rat feed (Vital Feeds); NC (normal control): non-diabetic rats that received the rat feed (Vital Feeds); LDH=Lactate dehydrogenase, ALP=Alkaline phosphatase, ALT=Alanine amino transferase, AST=Aspartate amino transferase, TP=total protein, ALB=Albumin.).

**Effect of FFFO on serum lipid profile of diabetic rats**

**Figure 1 (A-E):**
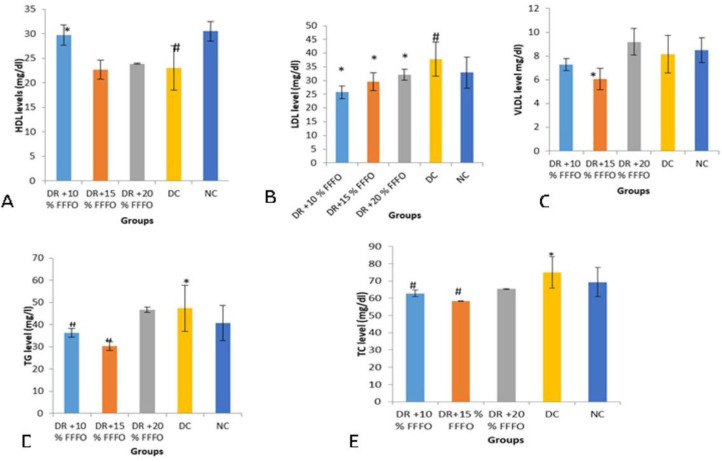
Effect of FFFO on lipid profile in diabetic rats. Values are mean±standard deviation (n=5) *p<0.05 shows a significant difference when compared with the diabetic control group (DC); #p<0.05 shows a significant difference when compared with the normal control group (NC). DR+10% FFFO: diabetic rats that received10% feed formulated with *Ficus ottoniifolia *(FFFO) leaves; DR+15% FFFO: diabetic rats that received 15% FFFO; DR+20% FFFO: diabetic rats that received 20% FFFO; DC (diabetic control): diabetic rats that received the rat feed (Vital Feeds), NC (normal control): non-diabetic rats that received the rat feed (Vital Feeds). A=HDL: high-density lipoprotein, B=LDL: low density lipoprotein, C=VLDL: very low-density lipoprotein, D=TG: triglyceride, E=TC: total cholesterol

Figure 1 (A-E) shows the impact of FFFO on lipid profile indices. The induction of DM in the rats produced significant (p<0.05) increases in LDL-cholesterol, TG and TC, whereas HDL-cholesterol was reduced significantly (p<0.05). Conversely, the consumption of FFFO markedly increased (p<0.05) HDL, but decreased LDL-c and TG as the concentration of the feed increased, compared to the diabetic control group.

**Effect of FFFO on serum liver function markers in diabetic rats**

Table 2 presents the results of the effect of FFFO on serum liver function markers in diabetic rats. The diabetic state induced significant (p<0.05) elevations in the serum activity of ALT and LDH, and levels of ALB compared to the normal control. Consumption of FFFO in group 1, 2 and 3 markedly (p<0.05) reduced the serum activities of LDH, ALP, ALT and TP as concentration of the feed increased, compared to the diabetic control. However, the serum activity of AST was elevated significantly (p<0.05) compared to the diabetic control.

## Discussion

The present study evaluated the antihyperglycemic, antihyperlipidemic and hepaprotective effect of FFFO in diabetic rats. Induction of DM in the rats caused significant increases in the blood glucose level. The elevated blood glucose in diabetic rats observed in this study, could be due to selective destruction of β cells of the pancreas by cytotoxic action of alloxan resulting in low levels of insulin or inability of the β cells to produce insulin, culminating in derangement in glucose homeostasis (Klinke, 2008 ▶). However, administration of FFFO to the diabetic rats decreased the elevated blood glucose and hence underscores its hypoglycemic effect (Table 1A). Attenuation of the elevated blood glucose level by FFFO in the diabetic rats is reflective of reversal of insulin resistance or increment ofthe secretion of insulin perhaps by regeneration of pancreatic β-cells destroyed by alloxan cytotoxic action in diabetic rats (Balamurugan et al., 2014 ▶). Flavonoids are known to act in the regeneration of damaged βcells in alloxan-induced diabetic rats and equally function as insulin secretagogues (Alagammal et al., 2012 ▶) and *F. ottoniifolia *leaves have been reported to be rich in carotenoids, flavonoids, phenols and tannins (Amaechi and Daniel, 2016 ▶) which have strong antioxidant activity that possibly will be accountable for the hypoglycemic efficacy found in this study. 

The observed weight loss in diabetic control rats relative to the normal control could be as a result of fat and protein catabolism or muscle wasting implicated in DM (Veeramani et al., 2008 ▶). The administration of FFFO caused gain in body weight of the rats in comparison to the diabetic control rats (Table 1B). The antihyperglycemic effect may inhibit the metabolic alterations associated with loss of body weight, hence the potential of FFFO to prevent reduction in weight and/or muscle wasting was observed. Additionally, the observed marked improvement in body weight of the rats after the administration of FFFO may be due to the good nutritional value and the stimulating potential of the feed on most aspects of carbohydrate metabolism, such as swift intake of glucose by the cells, accelerated rate of absorption from the gastrointestinal tract and enhancement of insulin secretion with its attendant secondary effects on carbohydrate metabolism (Guyton and Hall, 2000 ▶). 

In the present study, the experimental diabetes induced hyperlipidemia demonstrated by elevated levels of LDL, TG and TC, and considerable (p<0.05) reduction in HDL-cholesterol (Figure 1). Elevated TG in the diabetic rats may due to diminished clearance and accelerated synthesis of the main transporters of endogenously produced triglycerides (Rawi et al., 1998 ▶) and TC elevation might possibly be due to speeding up of intestinal cholesterol synthesis (O'Meara et al., 1990 ▶) or an increase in the rate at which cholesterol is absorbed from the intestine (Mathe, 1995 ▶). The significant increase in LDL cholesterol in diabetic rats may possibly be due to excessive production of VLDL by the hepatocytes or diminution in the clearance of VLDL and LDL from the circulation (Tsustsumi et al., 1995 ▶). Hypercholesterolemia and hypertriglyceridemia are associated with DM arising from increased hepatic lipolysis, adipose tissue fat mobilization and underutilization of glucose resulting from insulin absence or insensitivity (Ebrahimi et al., 2016 ▶). However, FFFO administration caused marked increment ofserum HDL-cholesterol and significant diminution in LDL, TG and TC relative to the diabetic control (Fig 1). 

The marked (p<0.05) elevation in the activity of ALT and non-significant increase in activities of ALP and AST triggered by the induced diabetes in rats (Table 2) is suggestive of liver injury (Otunola and Afolayan, 2015 ▶). The rise in the activities of ALT, ALP, AST and LDH in blood may be attributed to their leakage from the hepatic cells into the blood stream (Navarro et al., 1993 ▶). Elevation in the activity of serum ALT (p<0.05) and AST (p>0.05) on diabetes induction (Table 3), suggests hepatic injury, resulting in alteration of its structural architecture (Ubani et al., 2011 ▶). The administration of FFFO produced marked decreases (p<0.05) in the activity of alanine aminotransferase, aspartate aminotransferase and alkaline phosphatase as shown in Table 2, in comparison to the diabetic control. These suggest that FFFO has hepatoprotective potentials. 

In this study, there was a non-significant decrease in serum total proteins and albumin concentrations in the diabetic control in comparison with the non-diabetic control. The reduction in the concentration of total protein and albumin observed in the diabetic rats when compared to the non-diabetic ones, may be due to proteinuria, albuminuria, or accelerated degradation of protein which are biomarkers of diabetic nephropathy (Kaleem et al., 2008 ▶). However, treatment with the feed produced a non-significant improvement in albumin in DR+10% FFFO treated with 10% of the feed and in TP in DR+20%FFFO treated with 20% of the feed (Table 2). This effect may be possibly due to an increment in the insulin-mediated intake of amino acids, improvement of synthesis of protein and or retardation of protein catabolism (Ramachandran et al., 2012 ▶) 

The current study indicated for the first time, the antihyperglycemic potentials of rat-feed supplemented with *Ficus ottoniifolia *leaves. Our findings indicate that FFFO significantly decreased blood glucose level in the test rats. It equally mitigated other conditions secondary to hyperglycaemia such as hyperlipidaemia. The attenuation of ALT is suggestive that the feed may have protection against the liver. The beneficial health effects may be ascribed to the leaf phytoconstituents. Thus, further studies are required to isolate the bioactive compounds of the leaf that might be responsible for its glucose and lipid lowering potential.
